# Effects of a Concentric Rare-Earth Magnet on Menstrual Cycle Pain: A Parallel Group Randomized Pilot Study

**DOI:** 10.7759/cureus.12801

**Published:** 2021-01-20

**Authors:** Harvey Mayrovitz, Brittany Milo, Brooke Alexander, Marisa Mastropasqua, Yashaswani Moparthi

**Affiliations:** 1 Department of Medical Education, Nova Southeastern University Dr. Kiran C. Patel College of Allopathic Medicine, Davie, USA; 2 Department of Internal Medicine, Nova Southeastern University Dr. Kiran C. Patel College of Osteopathic Medicine, Davie, USA

**Keywords:** period-pain, menstrual pain, dysmenorrhea, magnetic therapy, static magnetic field

## Abstract

Background

Based on prior reports of the use of magnets to treat pain, our goal was to determine if a concentric rare-earth alternating-pole magnet reduced period pain versus a sham-magnet.

Methods

Participants were females (N=36, 18 to 35 years) who regularly experienced menstrual period pain ≥ six on the numeric pain rating scale (NPRS) of 0-10. Subjects were excluded if they took pain medication on the study day or had implanted pacemakers/metallic devices or secondary dysmenorrhea. Participants were randomized to wear a concentric neodymium-iron boron active-magnet (surface-field of 0.4 Tesla) or a sham magnet. The participant and investigator applying the device were blinded to the device used. The device was placed at the abdominal location of the reported greatest pain for 40-minutes, during which time the subject was able to conduct the normal activity. Pain scores were reported prior to device wearing and afterward. Participants with post-treatment NPRS ratings reduced by ≥ 35% from their pretreatment pain ratings were scored as having reduced pain; reductions < 35% were scored as no meaningful pain change. The threshold of 35% was chosen based on a survey of 10 women as to the level of pain reduction they viewed as meaningful to them. Of the 36 women in this pilot study, 19 wore an active-magnet and 17 wore a sham-magnet. Analyses were based on chi-square and Mann-Whitney statistical tests.

Results

Pre-treatment pain scores (mean ± SD) were similar for both groups. Magnet-vs-sham pre-treatment scores were, respectively, 7.16 ± 0.85 vs. 6.94 ± 1.20 (p=0.330). Corresponding median values for the magnet (N=19) and sham (N=17) groups respectively were seven pre-treatment and four post-treatment vs. six pre-treatment and six post-treatment. Post-treatment scores for magnet treated subjects (4.16 ± 2.20) were significantly less (p=0.027) than for sham-treated (5.53 ± 1.50). Of the 19 who wore a magnet, 11 experienced meaningful pain-reduction, and eight did not. Of the 17 who wore a sham, three experienced meaningful pain-reduction, and 14 did not. Magnet and sham wearing responses were statistically significant via chi-square analysis (chi-square=6.12, p=0.013). Percentage reduction in pain score was 41.8% ± 31.1% for magnet-treated vs. 20.8% ± 16.1%, for sham-treated (p<0.05).

Conclusions

Results suggest that short-term wearing of the magnet herein investigated, produces a meaningful menstrual-pain reduction in some women. Thus, further expanded research seems warranted to determine if longer wearing times result in even greater pain reductions.

## Introduction

The purpose of this study was to evaluate the potential effectiveness of a static magnetic field (SMF) of a concentric rare-earth alternating pole magnet to reduce dysmenorrhea (menstrual or period pain). The motivation for this investigation derives from prior work that reported various forms of magnetic field therapy to reduce or eliminate pain in a number of conditions [1-4]. These prior studies used SMF of magnets of various designs and material including multipolar designs [5]. Successful pain-related outcomes of SMF therapy have been reported for trigeminal pain [6], fibromyalgia [7], pain associated with oral procedures [2], post-polio pain [4], neuropathic pain [8], chronic pelvic pain in women [1], and musculoskeletal pain [9]. However, it is unknown if such magnetic therapy may have a beneficial effect in treating period pain. As a first step in investigating this possibility, with an eye toward further research in this area, the present randomized parallel group study was undertaken. 

Mechanisms by which SMF might be involved in reducing pain are unclear but various hypotheses have been put forward [10]. These include magnetic field related changes in blood flow variously reported to increase [11], decrease [12], or remain unchanged [13]. It has also been reported that pain reductions are linked to factors that affect inflammatory processes [1]. Others suggest alterations in pain thresholds are involved [9].

Although there is disagreement whether static magnetic fields can consistently mitigate pain, one study that focused on treating women with chronic pelvic pain appeared to result in significant pain reduction [1]. These researchers used magnets that had a concentric bipolar configuration and reported that subjects who wore them for four weeks noted a reduction in chronic pelvic pain. Additionally, a pilot study indicated that a static magnet was effective in reducing pain due to dysmenorrhea [14]. In part motivated by these prior reports, the present study was undertaken to investigate the potential for SMF related reduction of dysmenorrhea in young women. The underlying hypothesis to be tested is that women treated with an active multipolar magnet of a specific design would experience a greater pain reduction than women treated with a sham-magnet. 

Part of this research was submitted as an abstract to the Consortium for Excellence in Medical Education and was published in their abstract book in April 2020.

## Materials and methods

Subjects

This randomized, parallel design pilot study adheres to Consolidated Standards of Reporting Trials (CONSORT) guidelines. It was approved by the Nova Southeastern University Institutional Review Board as protocol 2018-614-NSU and registered with ClinicalTrials.gov with registration number NCT04539691 (retrospective registration). It was conducted starting in February 2019 and concluded in February 2020, Female subjects (N =36) within the age range of 18 to 35 were included in this study. Recruitment was done by posting Institutional Review Board-approved fliers around the Nova Southeastern University campus with a phone number to contact one of the investigators. Women were accepted for participation if they reported that they experienced regular periods with the pain of at least a six on a numeric scale of 0-10 scale, with 0 signifying no pain and 10 signifying the worst possible pain. They were excluded from participation if any of the following were true: they had a pacemaker, implanted wires or other metallic devices (including but not limited to a copper IUD, metallic mesh used for hernia repair, and metallic pins used in bone repairs), they had secondary dysmenorrhea, including endometriosis, fibroids, or pelvic inflammatory disease, or they took pain relief medications the day of the study visit. After investigators explained the study and answered their questions, each eligible volunteer signed an Institutional Review Board approved consent form.

This study was conducted in a double-blinded manner. Neither subjects nor co-investigators knew whether a magnet or sham was used on each subject. A list of random numbers was used to identify the magnets and shams. The list was only accessible to the principal investigator who compiled the data provided to him from the co-investigators. It was during the final analysis phase that subjects were classified as having received either active or sham magnets and group comparisons analyzed accordingly. 

Magnet and sham devices

**Figure 1:**
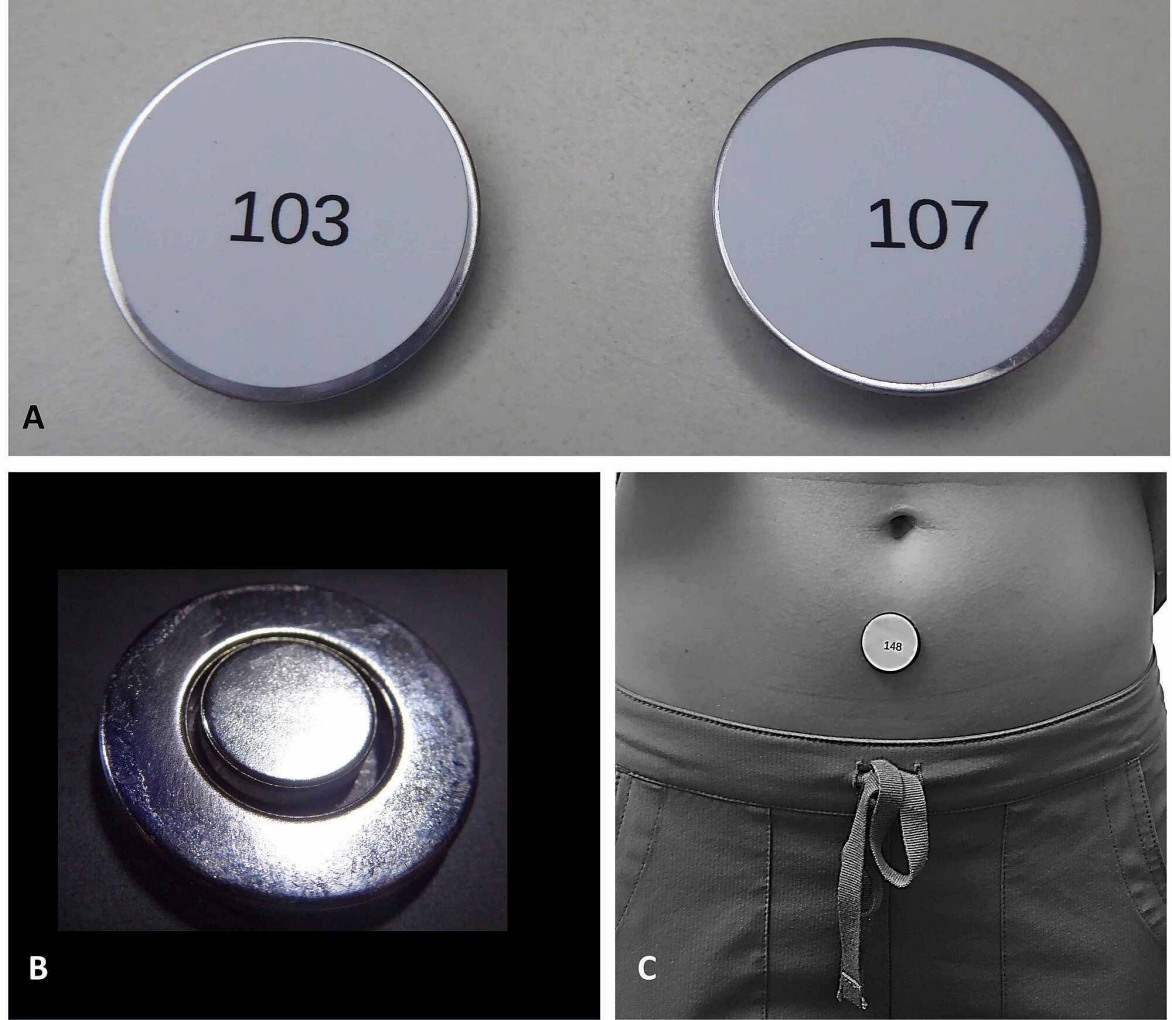
Magnet and sham devices Magnets and shams look similar and are randomly identified by number. (A) The device labeled as “103” is the magnet B. (B) A magnet shown without the covering. (C) Shown an example of placement of a device on a subject.

**Figure 2:**
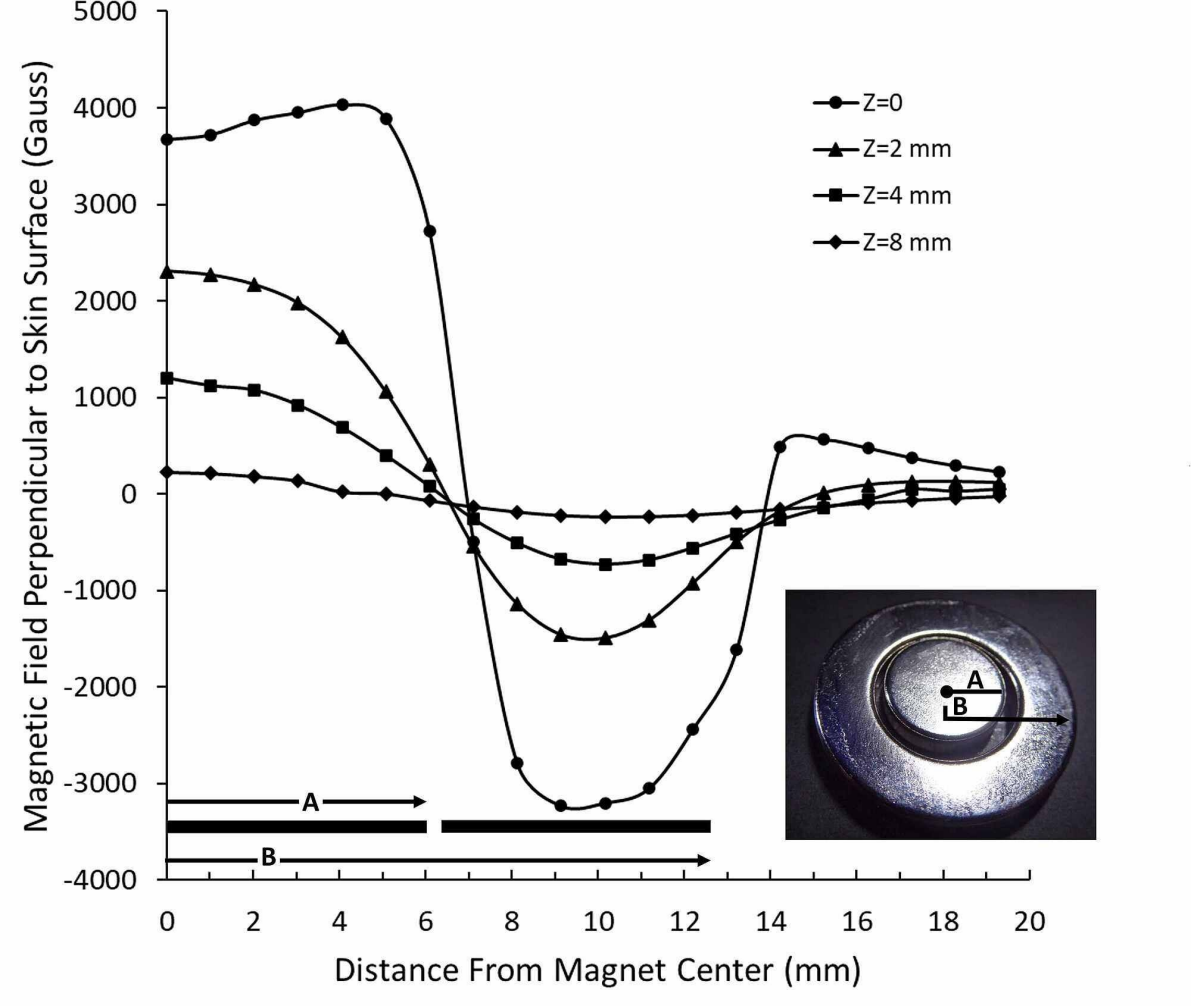
Field of the magnet Magnetic field perpendicular to the plane of the magnet is shown for the surface field (z=0) and for vertical distances of 2, 4, and 8 mm from the surface. The dimensions A and B correspond to the radius of the inner magnet and outer magnet, respectively, as illustrated in the inserted picture of the magnet.

The magnet and sham devices used visually appeared similar and had the same dimensions and weight. Each device was labeled with a unique number (Figure 1A) and was provided for use in this research study by Niiomed, Ft. Lauderdale, Florida, USA. The magnet (Neodymium-Iron-Boron) had a concentric design (Figure 1B) in which two concentric magnets produce a single magnetic array that, according to the manufacturer's descriptions, produces a greater magnetic flux per unit volume and can deliver better tissue penetration as compared with other magnet designs. This is stated to be achieved because the concentric design permits adjacent zones of polarity to mutually reinforce the magnetic field of one another resulting in increased intensity at the surface as described and illustrated in US patent # 9943699. The magnet employs a 12.7 mm diameter central element magnetically suspended in the core of a 25.4 mm outer diameter ring magnet, held in position via mutual magnetic attraction. An additional feature of the magnet used in this study relates to the use of a ferromagnetic backplate (1 mm thickness) providing enhanced magnetic field penetration. Since the magnetic flux from the two elements is mutually reinforcing surface field levels of up to 0.4T are achievable which is 50% greater than if the central core were of like polarity. The placement of a device on a subject is illustrated in Figure 1C. The magnetic field pattern of the magnet used is shown in Figure 2. The fields were measured every mm using the Kanetec Tesla meter, model TM-801EXP (Kanetec, Bensenville, IL, USA).

Procedures

**Figure 3:**
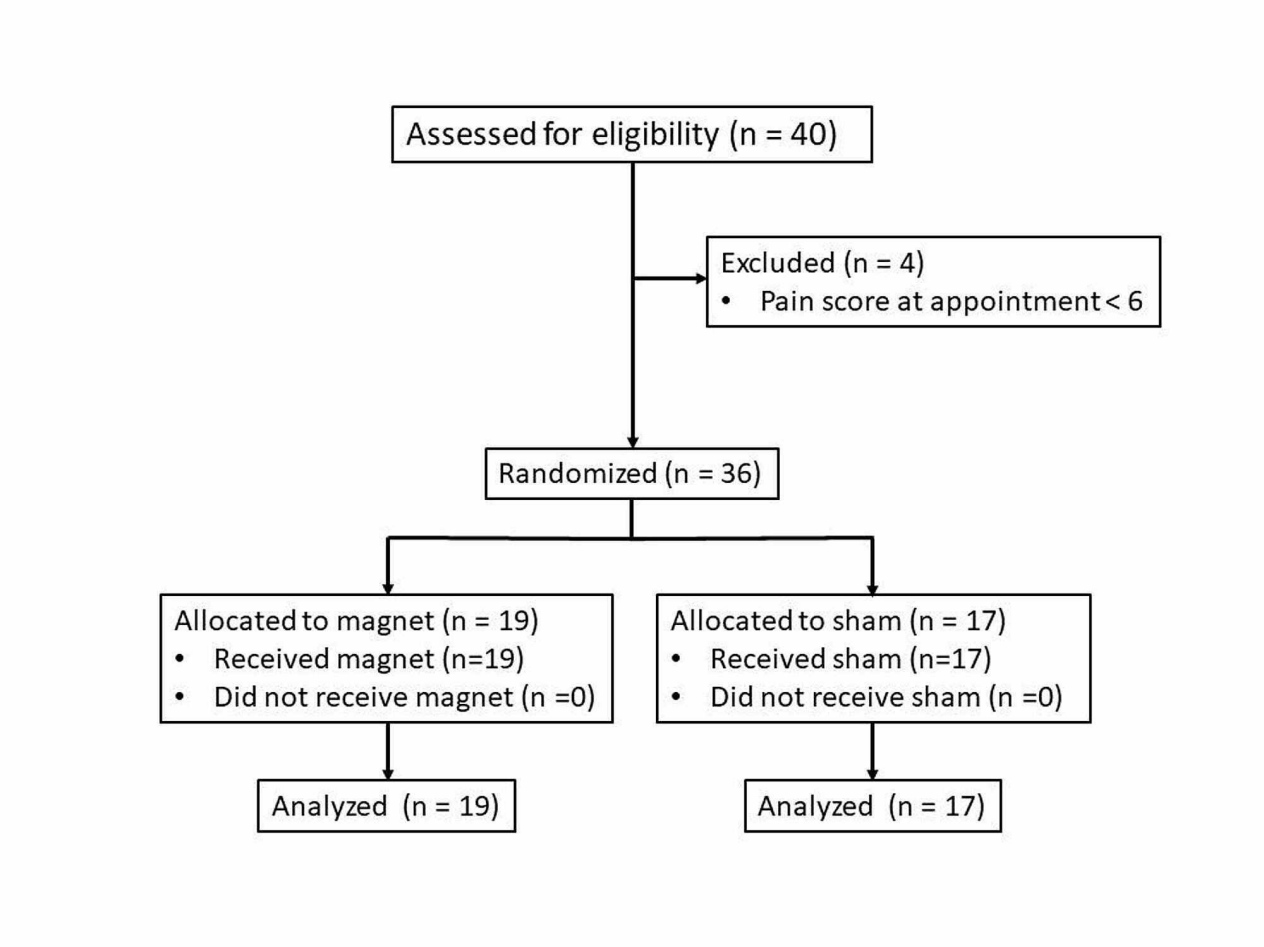
CONSORT type flow diagram CONSORT: Consolidated Standards of Reporting Trials

A CONSORT type flowchart showing an outline of the overall study procedures is shown in Figure 3. An experimental session was initiated when a subject experiencing menstrual pain called or texted a co-investigator. A meeting time/place was arranged at a time when the subject was feeling menstrual-related pain perceived as at least 6/10. This was communicated to one of the co-investigators via a text or phone message. At the meeting, the co-investigator verified the pain level once again. The subject was excluded from participation if the subject reported a pain level at that time that was not at least 6/10. This was true of four of the 40 potential subjects. For those satisfying the inclusion and exclusion criteria, demographic information, pain score, and description of the pain were recorded. With the aid of the subject, the site of pain was located as accurately as possible as the subject palpated the area while she was standing. Next, while the subject was standing, the co-investigator placed the magnet or sham device at the location designated by the subject. As noted, neither the subject nor the co-investigator knew whether a sham-magnet or active-magnet was being applied. A large number of devices, each within their own cardboard box, were labeled with numbers on the outside of the box and also on the device itself as illustrated in Figure 1. These boxes and number placements were provided to the investigators by the device manufacturer along with a coded list. The accuracy of the list with respect to whether it properly identified active magnets and shams was verified by the principal investigator who was not involved in either the recruitment or device placement process. An equal number of active and sham magnets so labeled were available for selection to use. The co-investigator, who was to place the device on the subject, would select one of the boxes at her whim while it was still in the box. This device was then affixed to the subject’s skin via a hypoallergenic adhesive backing. After device placement the subject was excused and able to perform daily activities except for running, exercise, and taking pain-relieving medications. The subject was advised to return to the lab at a certain time that represented a device wearing time of 40-minutes. Upon return, she reported her current pain level. The device was removed and the encounter concluded. Of 36 subjects who participated, 19/36 (52.8%) wore a magnet, and 17/36 (47.2%) wore a sham. All were included in the analysis phase.

Analysis

The primary data consisted of pain scores at two-time points; one prior to device exposure and one after 40-minutes of device wearing. Outcomes were determined by chi-square analysis of the number of subjects in whom pain was or was not meaningfully reduced. Subjects with pain scores reduced by ≥ 35% were scored as having meaningful pain reductions. Subjects with pain score reductions < 35% were scored as not having meaningful pain change. The choice of a threshold value of 35% pain reduction as a meaningful reduction was based on a pre-study survey of 10 women as to what minimum pain reduction, they would consider meaningful to them. Choices were 15%, 25%, 35%, 45%, 50%, or 60%. Eight of 10 chose 35%, one chose 25% and one chose 45%. Statistical significance of a difference in meaningful pain reduction as assessed via the chi-square analysis was based on an obtained p-value < 0.05. Group differences in absolute values of pain scores were test using the non-parametric Mann-Whitney test with a statistical significance difference judged on the basis of a p-value <0.05.

## Results

Entry pain levels (mean ± SD) for the groups were similar, with magnet and sham groups being respectively 7.16 ± 0.85 vs. 6.94 ± 1.20, (p = 0.330). Corresponding median values for the magnet (N=19) and sham (N=17) groups, respectively, were seven pre-treatment and four post-treatment vs. six pre-treatment and six post-treatment. Post-treatment scores for the magnet treated group were reduced to 4.16 ± 2.20 and 5.53 ± 1.50 for the sham-treated group which was significantly less of a pain score reduction (p = 0.027). Of the 19 subjects who wore a magnet, 11 experienced meaningful pain reduction according to the specified criterion, and eight did not. Of the 17 who wore a sham, three experienced meaning full pain reduction and 14 did not. The difference between magnet and sham wearing was statistically significant via chi-square analysis (chi-square = 6.12, p = 0.013). Percentage reduction in pain scores was 41.8% ± 31.1% for the magnet-treated group compared to 20.8% ± 16.1% pain reduction for those sham-treated. This difference was statistically significant (p = 0.50) based on the non-parametric Mann-Whitney test.

## Discussion

The present findings appear to be the first to provide some direct evidence that short-time wearing of the magnetic device used in this study might be useful to reduce the pain associated with significant menstrual pain. Such period pain has a variable prevalence between 16% and 91% with severe pain being measured in 2-29% of women [15]. Menstrual pain is most frequently treated by non-steroidal anti-inflammatory drugs and hormonal therapies [16]. These treatments have variable effectiveness [17] and are associated with side effects including gastrointestinal upset, renal injury, and cardiovascular events with NSAIDs and unscheduled bleeding, acne, migraines, weight gain, and mood disorders with hormonal therapies [18]. The present results demonstrated that on average more women experienced statistically significant pain reduction when wearing the active magnet, however, some women also experienced similar levels of pain reduction when wearing the sham-magnet. Further, some women who wore the magnet for the 40-minute duration experienced no pain reduction. Thus, it would appear that, as with many forms of therapy, its effectiveness is dependent on other factors that could not be further illuminated with the current study design. It could be argued that one of those factors is the duration of wearing. The selected time interval of 40-minutes of wearing in this study was somewhat arbitrary and chosen in part for logistical reasons and less based on prior SMF pain-related data. There is a body of literature in which pulsed electromagnetic fields (PEMF) have been used for pain alleviation and many of those protocols utilize a treatment duration between 30-60 minutes [19]. The present duration of treatment (40-minutes) was chosen in part based on this observation. It is unclear whether a longer duration would have yielded even more favorable results and remains to be evaluated.

As noted in the introduction, other workers have used SMF to try to reduce pain in a variety of conditions but most of these have utilized longer durations of application. Successful treatment of chronic pelvic pain was reported to occur after four-weeks of treatment [1]. It has also become reasonably clear that the effectiveness of SMF treatment for pain may depend on the specific underlying condition.

For example, SMF exposure significantly reduced pain perception in a group with temporomandibular disorders, but not in groups with alveolitis or aphtha [2]. Other studies have demonstrated that physiologic effects of the magnet, such as increased blood flow, may not last long after the magnet is removed [11]. Some studies have reported no significant effects of SMF therapy. Examples include osteoporosis-related pain [3], diabetic-related neuropathic pain [20], pain associated with surgical incisions [21], nonspecific foot pain [22], plantar heel pain [23], chronic pelvic pain syndrome, and rheumatic pain in patients wearing magnetic bracelets with peak field strengths of about 200mT [24]. It is unclear if these specific negative outcomes relate to fundamental processes or more to the specific commercial devices used [25], the experimentally induced acute pain model that was evaluated [19], the specific condition being treated, or the parameters of the treatment field. It may also be true that SMF therapy is useful in only some conditions for which these were not a part [26].

The present study was double-blind and placebo-controlled that focused specifically on dysmenorrhea pain with a consistent SMF intensity over a fixed 40-minute interval yielding positive results with regard to pain reduction. As such, the SMF from this magnet type may be considered a possible alternative to traditional pain management such as drugs in amenorrhea. Current treatment guidelines for dysmenorrhea include non-steroidal anti-inflammatory drugs (NSAIDs) and hormonal contraceptives [16]. The magnet could be especially useful in women who are unable or unwilling to take medication or as a non-side effect substitute to traditional NSAIDs and contraceptives. The use of NSAIDs is associated with gastrointestinal, renal, and cardiovascular effects [18], and oral contraceptives are associated with numerous side effects such as nausea, fluid retention, vaginal discharge, breakthrough bleeding and venous thromboembolic disease [27], and also numerous contraindications such as drug interactions or history of gynecological cancer [28]. To date, SMF has no known adverse effects and can be a safe alternative to commonly prescribed medications. Unlike the current pharmacological or invasive methods described above, the SMF induces the body’s natural mechanisms of reducing pain instead of using medications, hormones, or implantable devices. 

In addition to the typical approaches listed, there are a number of holistic approaches that have been studied to aid in reducing dysmenorrhea. One study completed in 2014 determined that the use of heat, transcutaneous electrical nerve stimulation and yoga are effective for relief of primary dysmenorrhea [29]. Another clinical trial reports that heat therapy, exercise, and self-delivered acupressure have a significant reduction in pain as well [30]. In future studies, these holistic methods can be combined with the use of an SMF to determine if combining these methods proves to be the most effective while limiting side effects. 

Because of the holistic component of using SMF, researchers will be enticed to further explore the use of SMF to treat dysmenorrhea or even other visceral pain, and this study provides a framework and background for further research into menstrual pain and its treatment. Future studies could incorporate exercising and other non-pharmacological methods of pain reduction in combination with the magnet to assess the effectiveness of pain reduction. Other studies can focus on the effect that the location of the magnet has on the effectiveness of the treatment.

There are several limitations to this study. Although the number of subjects included was not large it was likely sufficient for a pilot study as herein conducted. A specific limitation was that the duration of pain reduction following removal of the device was not assessed. Further, only one duration of magnet wearing time was used and it is unknown if a longer wearing time would result in greater pain reduction. Another potential limitation was that there was sole reliance on the perception of pain reported by the subject. However, this is a standard practice in pain studies. In addition, placebo effects are not likely but cannot be fully ruled out. The subjects were queried as to whether they had touched or otherwise probed the device they were wearing. All replied in the negative. However, they were not under direct observation during their wearing-time. Additionally, we do not know if the non-responders were due to a “distance-to-target” increase due to excess weight. Given the measured penetration depth of the magnet, it is not likely that excess fat would have altered the potential magnetic effect if present.

## Conclusions

The results of this study suggest that short-term wearing of a magnet of the design and the field properties investigated may provide adequate relief of menstrual pain in some women. Though the sample size of this study was relatively small, it provides a solid foundation for further research into the benefits of magnetic therapy for dysmenorrhea. Using a similar study design, further research can be conducted with alteration of certain variables. For example, the duration of time for which a participant wears a magnet may be increased to determine if longer use provides greater pain relief. Additionally, the use of magnetic therapy may be combined with traditional treatment modalities, such as non-steroidal anti-inflammatories, to determine if there is an additive effect on pain alleviation. Increasing the sample size for further studies would also provide insight into how effective magnet therapy is in the actual population. If the results of this study hold true, magnetic therapy may become a commonly used alternative to traditional pain management for dysmenorrhea.

## References

[1] Brown CS, Ling FW, Wan JY, Pilla AA (2002). Efficacy of static magnetic field therapy in chronic pelvic pain: a double-blind pilot study. Am J Obstet Gynecol.

[2] Laszlo JF, Farkas P, Reiczigel J, Vago P (2012). Effect of local exposure to inhomogeneous static magnetic field on stomatological pain sensation - a double-blind, randomized, placebo-controlled study. Int J Radiat Biol.

[3] Meszaros S, Tabak AG, Horvath C, Szathmari M, Laszlo JF (2013). Influence of local exposure to static magnetic field on pain perception and bone turnover of osteoporotic patients with vertebral deformity - a randomized controlled trial. Int J Radiat Biol.

[4] Vallbona C, Hazlewood CF, Jurida G (1997). Response of pain to static magnetic fields in postpolio patients: a double-blind pilot study. Arch Phys Med Rehabil.

[5] Duyvendak W (2007). Spinal cord stimulation with a dual quadripolar surgical lead placed in general anesthesia is effective in treating intractable low back and leg pain. Neuromodulation.

[6] Zhu Y, Wang S, Long H, Zhu J, Jian F, Ye N, Lai W (2017). Effect of static magnetic field on pain level and expression of P2X3 receptors in the trigeminal ganglion in mice following experimental tooth movement. Bioelectromagnetics.

[7] Paolucci T, Piccinini G, Iosa M (2016). Efficacy of extremely low-frequency magnetic field in fibromyalgia pain: a pilot study. J Rehabil Res Dev.

[8] Eccles NK (2005). A critical review of randomized controlled trials of static magnets for pain relief. J Altern Complement Med.

[9] Khoromi S, Blackman MR, Kingman A (2007). Low intensity permanent magnets in the treatment of chronic lumbar radicular pain. J Pain Symptom Manage.

[10] Pilla AA (2013). Nonthermal electromagnetic fields: from first messenger to therapeutic applications. Electromagn Biol Med.

[11] Yan Y, Shen G, Xie K (2011). Wavelet analysis of acute effects of static magnetic field on resting skin blood flow at the nail wall in young men. Microvasc Res.

[12] Mayrovitz HN, Groseclose EE (2005). Effects of a static magnetic field of either polarity on skin microcirculation. Microvasc Res.

[13] Mayrovitz HN, Groseclose EE, Markov M, Pilla AA (2001). Effects of permanent magnets on resting skin blood perfusion in healthy persons assessed by laser Doppler flowmetry and imaging. Bioelectromagnetics.

[14] Eccles NK (2005). A randomized, double-blinded, placebo-controlled pilot study to investigate the effectiveness of a static magnetic to relieve dysmenorrhea. J Altern Complement Med.

[15] Ju H, Jones M, Mishra G (2014). The prevalence and risk factors of dysmenorrhea. Epidemiol Rev.

[16] Burnett M, Lemyre M (2017). No. 345-primary dysmenorrhea consensus guideline. J Obstet Gynaecol Can.

[17] Oladosu FA, Tu FF, Hellman KM (2018). Nonsteroidal antiinflammatory drug resistance in dysmenorrhea: epidemiology, causes, and treatment. Am J Obstet Gynecol.

[18] Harirforoosh S, Asghar W, Jamali F (2013). Adverse effects of nonsteroidal antiinflammatory drugs: an update of gastrointestinal, cardiovascular and renal complications. J Pharm Pharm Sci.

[19] Fernandez MI, Watson PJ, Rowbotham DJ (2007). Effect of pulsed magnetic field therapy on pain reported by human volunteers in a laboratory model of acute pain. Br J Anaesth.

[20] Wrobel MP, Szymborska-Kajanek A, Wystrychowski G (2008). Impact of low frequency pulsed magnetic fields on pain intensity, quality of life and sleep disturbances in patients with painful diabetic polyneuropathy. Diabetes Metab.

[21] Cepeda MS, Carr DB, Sarquis T, Miranda N, Garcia RJ, Zarate C (2007). Static magnetic therapy does not decrease pain or opioid requirements: a randomized double-blind trial. Anesth Analg.

[22] Winemiller MH, Billow RG, Laskowski ER, Harmsen WS (2005). Effect of magnetic vs sham-magnetic insoles on nonspecific foot pain in the workplace: a randomized, double-blind, placebo-controlled trial. Mayo Clin Proc.

[23] Winemiller MH, Billow RG, Laskowski ER, Harmsen WS (2003). Effect of magnetic vs sham-magnetic insoles on plantar heel pain: a randomized controlled trial. JAMA.

[24] Leippold T, Strebel RT, Huwyler M, John HA, Hauri D, Schmid DM (2005). Sacral magnetic stimulation in non-inflammatory chronic pelvic pain syndrome. BJU Int.

[25] Szemerszky R, Szabolcs Z, Bogdany T, Janossy G, Thuroczy G, Koteles F (2018). No effect of a pulsed magnetic field on induced ischemic muscle pain. A double-blind, randomized, placebo-controlled trial. Physiol Behav.

[26] Carter R, Aspy CB, Mold J (2002). The effectiveness of magnet therapy for treatment of wrist pain attributed to carpal tunnel syndrome. J Fam Pract.

[27] Wong CL, Farquhar C, Roberts H, Proctor M (2009). Oral contraceptive pill for primary dysmenorrhoea. Cochrane Database Syst Rev.

[28] Williams RS (1992). Benefits and risks of oral contraceptive use. Postgrad Med.

[29] Kannan P, Claydon LS (2014). Some physiotherapy treatments may relieve menstrual pain in women with primary dysmenorrhea: a systematic review. J Physiother.

[30] Armour M, Smith CA, Steel KA, Macmillan F (2019). The effectiveness of self-care and lifestyle interventions in primary dysmenorrhea: a systematic review and meta-analysis. BMC Complement Altern Med.

